# Monte Carlo Analysis of Neck Linker Extension in Kinesin Molecular Motors

**DOI:** 10.1371/journal.pcbi.1000980

**Published:** 2010-11-04

**Authors:** Matthew L. Kutys, John Fricks, William O. Hancock

**Affiliations:** 1 Department of Bioengineering, The Pennsylvania State University, University Park, Pennsylvania, United States of America; 2 Department of Statistics, The Pennsylvania State University, University Park, Pennsylvania, United States of America; University of California, Irvine, United States of America

## Abstract

Kinesin stepping is thought to involve both concerted conformational changes and diffusive movement, but the relative roles played by these two processes are not clear. The neck linker docking model is widely accepted in the field, but the remainder of the step – diffusion of the tethered head to the next binding site – is often assumed to occur rapidly with little mechanical resistance. Here, we investigate the effect of tethering by the neck linker on the diffusive movement of the kinesin head, and focus on the predicted behavior of motors with naturally or artificially extended neck linker domains. The kinesin chemomechanical cycle was modeled using a discrete-state Markov chain to describe chemical transitions. Brownian dynamics were used to model the tethered diffusion of the free head, incorporating resistive forces from the neck linker and a position-dependent microtubule binding rate. The Brownian dynamics and chemomechanical cycle were coupled to model processive runs consisting of many 8 nm steps. Three mechanical models of the neck linker were investigated: Constant Stiffness (a simple spring), Increasing Stiffness (analogous to a Worm-Like Chain), and Reflecting (negligible stiffness up to a limiting contour length). Motor velocities and run lengths from simulated paths were compared to experimental results from Kinesin-1 and a mutant containing an extended neck linker domain. When tethered by an increasingly stiff spring, the head is predicted to spend an unrealistically short amount of time within the binding zone, and extending the neck is predicted to increase both the velocity and processivity, contrary to experiments. These results suggest that the Worm-Like Chain is not an adequate model for the flexible neck linker domain. The model can be reconciled with experimental data if the neck linker is either much more compliant or much stiffer than generally assumed, or if weak kinesin-microtubule interactions stabilize the diffusing head near its binding site.

## Author Summary

Kinesin molecular motors provide a valuable model for uncovering the interplay between nanoscale mechanics and biochemistry at the level of single protein molecules. The mechanism by which kinesin motors “walk” along microtubules involves both conformational changes in the motor domains, or “heads”, as well as diffusive movements in which one head searches for its next binding site on the microtubule. This diffusive search is constrained by the 14 amino acid neck linker domain, which must be sufficiently flexible to allow the free head to diffuse forward, yet sufficiently stiff to enable mechanical communication to the rest of the molecule. We have modeled this diffusive search and integrated it into a stochastic model of the kinesin chemomechanical cycle. We find that modeling the neck linker as a Worm-Like Chain, the model most frequently used to describe unstructured polypeptide chains, results in motor behavior that conflicts with published experimental results for kinesins containing naturally or artificially extended neck linker domains. These results suggest that either the mechanical properties of the neck linker domain must be fundamentally reevaluated or that there are motor-microtubule interactions that stabilize the motor domain at its next binding site.

## Introduction

Motor proteins in the kinesin superfamily are molecular machines that use the energy derived from ATP hydrolysis to transport organelles and other cellular cargo along microtubules. The 14 kinesin families are structurally diverse and display differences in motor velocity, directionality, and processivity that relate to their various cellular functions [1], [2]. Kinesin-1, (conventional kinesin), contains two 110 kDa heavy chains that consist of the N-terminal motor head, the flexible neck linker domain, the coiled-coil stalk, and the C-terminal cargo-binding tail. The primary cellular function of Kinesin-1 is the long distance transport of vesicles and organelles in neurons. Kinesin-1 is a processive motor, meaning it takes many steps of roughly 8 nm along the microtubule without detaching. This processive behavior requires coordination between the chemomechanical cycles of the two heads, such that at least one motor head remains attached to the microtubule at any given point in the cycle [3], [4].

The Kinesin-1 neck linker, a 14 amino acid domain that connects the globular motor head to the coiled-coil dimerization domain, has been the subject of intense experimental and theoretical investigations. This neck linker domain is thought to transition from a conformationally flexible unstructured state to a structured and docked state upon ATP binding, providing the principal conformational change in the motor [5], [6]. This neck linker docking provides a forward (plus-ended) bias to the motor and enables the free tethered head to diffuse to the next binding site approximately 8 nm away. Importantly, during this diffusive search, the neck linker serves as a tether that constrains the search of the motor head for the next microtubule binding site and ensures that that lateral or backward steps are exceedingly rare [7], [8]. Furthermore, when both heads are simultaneously bound to the microtubule, the neck linker needs to be sufficiently stiff that mechanical forces can be transmitted between the head domains to enable mechanochemical coordination between the two head domains [4], [9]–[11]. Hence, there are two competing design pressures – the neck linker must be sufficiently extensible to enable diffusional search of the tethered head for its next binding site, but it must be sufficiently stiff to transmit forces between the heads when both heads are bound to the microtubule.

To understand the dynamics of tethered diffusion in the kinesin walking mechanism, we have created a model of kinesin stepping that incorporates both chemical kinetics of the kinesin hydrolysis cycle and Brownian dynamics to represent the diffusion of the free motor head tethered by its flexible neck linker segment. The diffusion of the free head is modeled using a position-dependent stochastic differential equation where the drift (i.e. potential) is determined by the current chemical state of the motor, similar to a Brownian or flashing ratchet [12]. The mechanical properties of the neck linker domain play a central role in determining the diffusional characteristics of the free motor head, but its small size complicates experimental characterization. Hence, we have chosen to keep the diffusional model intentionally simple so as to minimize the number of assumptions and have used the model to test different mechanical representations of the flexible neck linker domain. Hyeon and Onuchic previously used a computational approach based on crystal structures of kinesin and tubulin to explore the dynamics of tethered head binding to the microtubule, but they did not explicitly investigate the role that neck linker mechanics play in this diffusive search [13]. The Brownian dynamics approach used here is similar to that of Atzberger et al. [14], with the difference that we have focused on a one dimensional model to highlight the role of different models for the neck linker and have expanded the chemical hydrolysis cycle to better account for the current state of the field.

**Figure 1:**
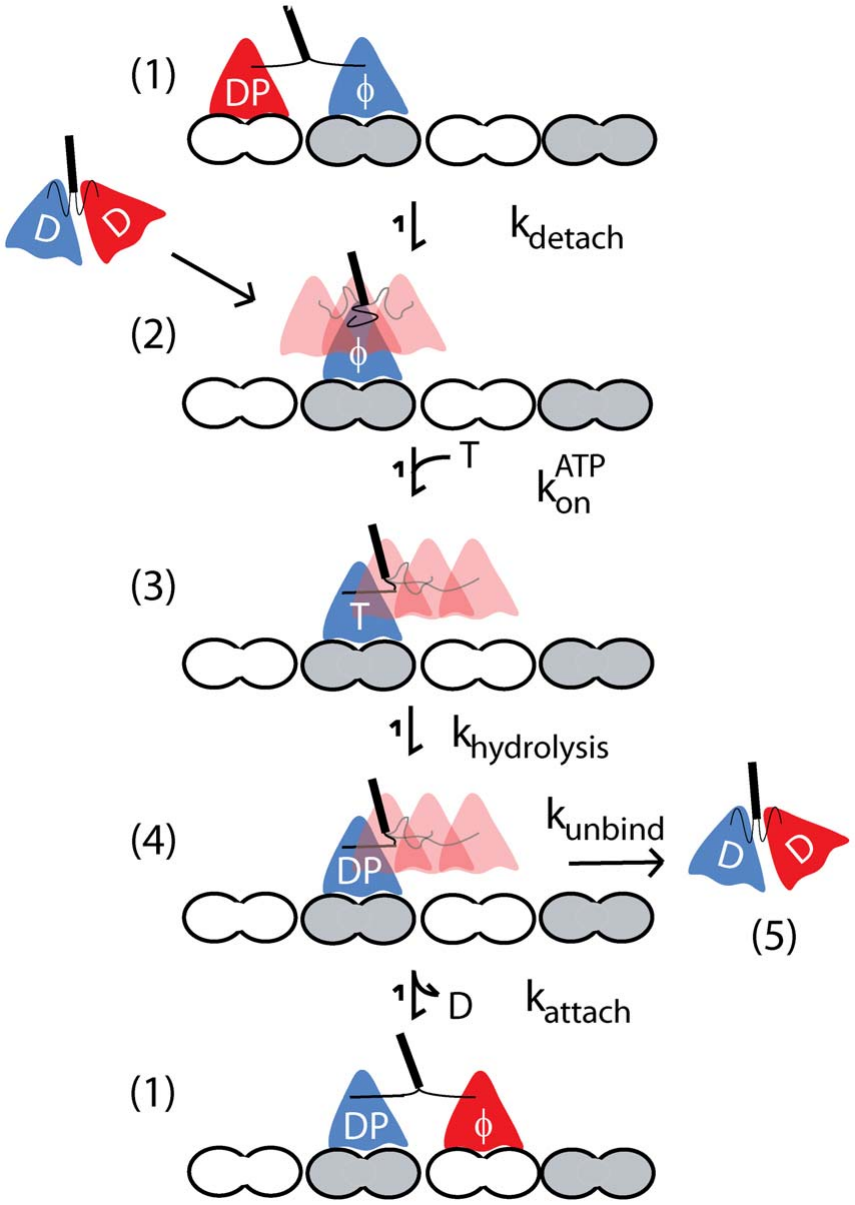
Kinesin Chemomechanical Pathway. Working model for the Kinesin-1 chemomechanical pathway based on previous experimental work. Nucleotide abbreviations are as follows: T = ATP, D = ADP, DP = ADP.Pi, φ = No nucleotide. For clarity, ADP bound to tethered head in states 2–4 is not shown. In State 2 the tethered head diffuses, tethered by both neck linker domain, while in states 3 and 4 the neck linker domain of the bound head is docked, leading to a displacement of the tethered head towards the next binding site. State 5 represents motor detachment. Note that the number of steps per interaction (motor processivity) can be approximated by k_attach_/k_unbind_.

The kinetic model for the Kinesin-1 hydrolysis cycle that underlies this work is presented in Figure 1. This model is built on a large body of kinesin biophysical and biochemical studies [3], [4], [6], [15], [16] and was recently used to investigate differences between Kinesin-1 and Kinesin-2 motors [17], [18]. In the model the motor starts in State 2 with one head bound and the tethered head freely diffusing and able to bind to either the next binding site on the microtubule or its previous binding site. ATP binding causes ordering of the neck linker domain and displacement of the tethered head toward the plus-end of the microtubule (State 3). Following ATP hydrolysis (State 4), the tethered head diffusively searches for the next binding site and binds there (State 1) or, if this attachment is too slow the bound head releases from the microtubule (State 5), terminating the run. By incorporating rate constants into a standard Markov stepping model, this model was able to reproduce Kinesin-1 velocity and processivity characteristics across a range of ATP concentrations [9], [17].

A principal motivation of the present study is to understand how extending the kinesin neck linker alters kinesin stepping behavior. The consensus from structural studies is that for Kinesin-1 to take an 8 nm step, the neck linker must extend a distance approaching its full contour length [10], [19], [20]. Interestingly, sequence analysis suggests that diverse kinesins that carry out quite different transport functions in cells and have considerably different motor properties from Kinesin-1 possess longer neck linkers [21]. We recently showed that Kinesin-2 motors, which have a 3 amino acid insertion in their neck linker are less processive than Kinesin-1 motors [17]. We then went on to show that extending the 14 amino acid Kinesin-1 neck linker decreases motor processivity considerably and shortening the 17 amino acid Kinesin-2 neck linker enhances processivity, while motor velocity is only weakly correlated with neck linker length [18]. These results are essentially consistent with recent studies from three other labs, with discrepancies largely accounted for by differences in experimental methodology [8], [22], [23]. While it is clear that extending the neck linker reduces motor processivity, what is not clear is which step or steps in the kinesin chemomechanical cycle are altered. As can be seen in Figure 1, the probability that a motor detaches during each step is controlled by a race between binding of the tethered head to the next binding site (State 4 to State 1 transition, k_attach_) versus unbinding of the bound head (State 4 to State 5 transition, k_unbind_). Hence, any perturbation that alters the rate that the tethered head binds to the microtubule is expected to alter motor processivity. Because tethered head binding involves diffusion of the head to the next binding site, followed by tight binding and ADP release, any constraints on this diffusional search imposed by the mechanical properties of the neck linker domain are expected to have a strong effect on motor processivity. The goal of the present simulations is to use the constraints provided by the experimental data to better understand the mechanical properties and dynamic behavior of the kinesin neck linker domain.

**Figure 2:**
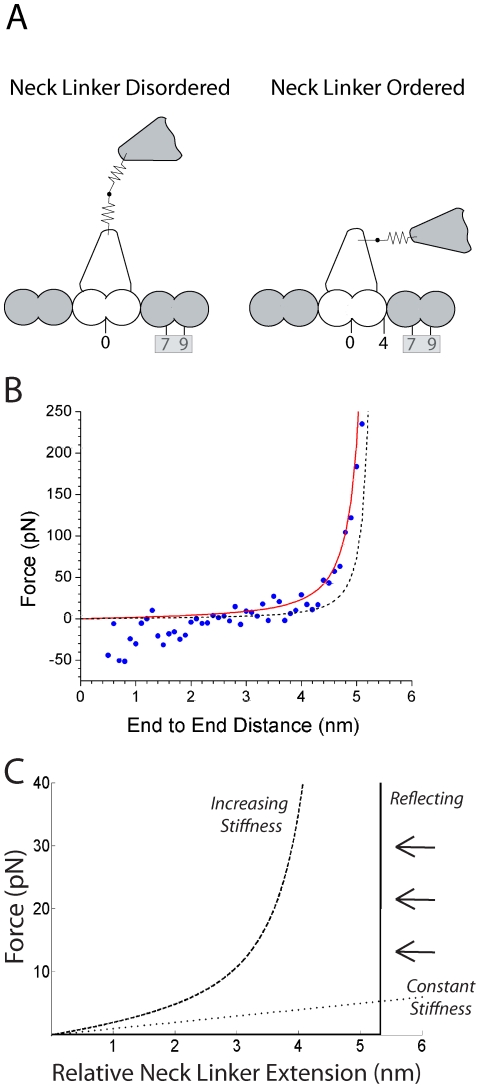
Kinesin Structural Models. A: Comparison of neck linker structures before and after docking. In state preceding ATP binding (left) the tether between the heads consists of both neck linkers (28 amino acids) with no forward bias (initial position 0 nm). Upon nucleotide binding (right), the rear neck linker docks to its motor domain, providing a 4.1 nm bias toward the microtubule plus-end. At this point the free motor head is tethered only by its 14 amino acid neck linker. The microtubule binding zone (7.2–9.2 nm, grey box) is defined as a region within 1 nm of the binding site. The motor is also permitted to bind to a site 8.2 nm to the rear (not shown), but this rarely occurs. B: Kinesin-1 force-extension profile from molecular dynamics simulations. Solid line shows fit to WLC with *L_p_* = 0.7 nm and dashed line shows fit to WLC with *L_p_* = 2 nm; both use *L_c_* of 0.364 nm per amino acid as described in text. Molecular dynamics results adapted from Hariharan and Hancock [21]. C: Force extension profiles of the neck linker domain shown for the *Increasing Stiffness Model* (dashed line), *Constant Stiffness Model* (dotted line) and *Reflecting Model* (solid line). Arrows represent the reflecting barrier characteristic of the *Reflecting Model*.

In the present study, we examine the tethered diffusion of the kinesin head under three different qualitative regimes, corresponding to three mechanical representations of the neck linker domain. Each of these approaches constrains the diffusion about a central point through a restoring force that depends on the current chemical state of the motor, but the nature of the restoring force differs (Figure 2). The *Constant Stiffness Model* is analogous to a simple Hookean spring, in which the restoring force is proportional to the distance from the center point. The *Increasing Stiffness Model* is qualitatively similar to a Worm-Like Chain (WLC) entropic spring, in which the restoring force increases nonlinearly with extension. The Worm-Like Chain is the most common model used to describe the force-extension properties of unstructured polypeptides, and both AFM experiments [24], [25] and Molecular Dynamics simulations [21] provide evidence that it is a reasonable approximation of neck linker mechanics. Finally, the *Reflecting Model* consists of a compliant Hookean spring up to a maximum contour length where the restoring force is infinite. Surprisingly, the *Increasing Stiffness Model* simulations do not agree well with experimental data, while the *Reflecting Model* simulations do agree with experiments. These results suggest that the Worm-Like Chain may not be an appropriate description of Kinesin-1 neck linker mechanics or at least must be modified from its current form to accurately describe the diffusive tethering of the free motor head. Alternatively, the results can be explained by positing a weak-binding state that stabilizes the tethered head near its binding site on the microtubule.

## Results

In the present work, we investigate the effect of extending the kinesin neck linker on the tethered diffusion of the free motor head using a model that incorporates a substantial amount of biological detail but a minimal number of assumptions. The motor domain is approximated as a sphere with a diameter of 6 nm [26], and its diffusion is modeled in one dimension along a lattice of binding sites spaced 8.2 nm apart (the spacing of tubulin dimers along a microtubule protofilament). Thermally-driven diffusion of the free kinesin head is constrained by the flexible neck linker domain, and binding to the microtubule is allowed only when the head is within ±1 nm of the next binding site on the microtubule (Figure 2A). ATP binding to the bound motor head (State 3 in Figure 1) is thought to promote docking of the neck linker domain [5], [6], which can be intuitively described as a diffusion and stabilization rather than the rigid powerstroke of the myosin lever arm. This neck linker docking is incorporated into the model by switching the tethered diffusion from a center point position of zero and a tether length equal to both neck linker domains (State 2 in Figure 1) to a center point position of 4 nm toward the microtubule plus-end and a tether consisting of only one neck linker (Steps 3 and 4 in Figure 1). Completion of a step requires diffusion of the tethered head to the next binding site followed by attachment and ADP release (State 1). As described below, this straightforward model challenges the assumption that diffusion and binding is rapid and unconstrained.

### Increasing Stiffness Model

Polymers such as DNA and unfolded polypeptides are often described as “entropic springs” because stretching them, which reduces their number of possible conformational states, requires energy input to compensate for the loss of entropy [27]. From the WLC formalism, the force, *f_WLC_(x)*, required to extend a polypeptide chain an end-to-end distance *x* is given as [28],[29]:
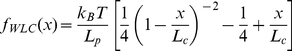
(1)where, *k_B_T* is the Boltzmann constant times the absolute temperature, *L_p_* is the persistence length, and *L_c_* is the contour length of the polymer. The persistence length of unstructured polypeptide chains has been measured to be in the range of 0.5 to 2 nm [24], [25], [30], [31], though the sequence dependence and the degree to which these measurements extrapolate to chains as short as 14 residues are not clear. We recently carried out molecular dynamics simulations to measure the force-extension properties of the Kinesin-1 neck linker domain [21]. The results of these simulations are replotted in Figure 2B along with a curves for a WLC model with *L_p_* of 0.7 nm, which accounts well for the data, and a *L_p_* of 2 nm, which is less able to account for the data, and a contour length, *L_c_*, of 0.364 nm per amino acid. Most studies in the literature [24], [25], [30], [31] use a contour length of 0.38 nm per amino acid, which is the dimension of a single amino acid residue [32]. However, this value ignores the bond angle between adjacent amino acids, which, when taken into account yields a *L_c_* of 0.364 nm per amino acid [33]. Because this improved value gave better fits to our molecular dynamics data, all of our *Increasing Stiffness Model* simulations used *L_c_* = 0.364 nm per amino acid and *L_p_* of 0.7 nm or, for comparison an *L_p_* of 2 nm.

The position of the tethered head, *X(t)*, was computed using the overdamped Langevin equation comprising viscous forces, tethering by the neck linker domain, and Brownian forces on the head domain. Mathematically, this was expressed as:
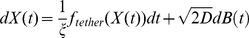
(2)where *ξ* is the friction coefficient, *f_tether_* is the force of the tethering neck linker domain, *D* is the diffusion constant of the head domain and *B(t)* is a Wiener process representing Brownian motion of the head (see Methods for further details) [34], [35]. Numerical solutions to the Langevin equation under the *Increasing Stiffness Model* were obtained using the Euler method [36].

**Figure 3:**
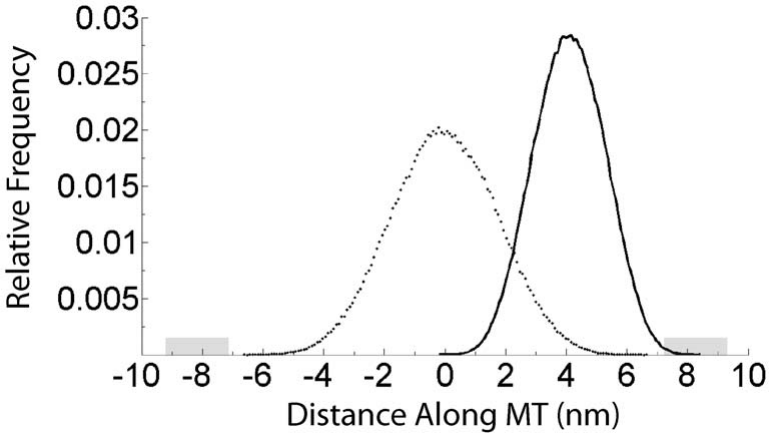
Stationary Distribution Profile of Tethered Head. Stationary positional distribution of the tethered Kinesin-1 motor domain during its diffusive search using the *Increasing Stiffness* neck linker model. Dotted line shows state before ATP binding (State 2 in Figure 1) where both neck linkers are disordered and there is no positional bias of the tethered head. Solid line shows state following ATP binding (States 3 and 4 in Figure 1) where docking of one neck linker causes a 4 nm displacement toward the microtubule plus-end and diffusion is tethered by remaining neck linker. Note that before neck linker docking the free head cannot reach the next microtubule binding site (grey zone), while after neck linker docking the free head spends only a small fraction of the time (<1%) near the binding site.

To explore the different elasticity models in greater detail, Brownian dynamics simulations were performed to obtain stationary distributions of the motor head during the diffusive search. While these stationary distributions are only suggestive of phenomena in the full model where transient behavior can be a factor, they can provide insight into the behavior of the competing models. Figure 3 shows that in no-nucleotide states where both neck linkers are disordered and there is no positional bias of the free head, thermal motion is insufficient to achieve either forward or rearward binding of the free head. Characteristic of the *Increasing Stiffness Model*, when the free head diffuses more than a few nanometers away from its resting position, the restoring forces rise dramatically, preventing further progress. In contrast, following ATP binding, which docks one neck linker and provides a 4 nm forward displacement bias, the free motor head is able to diffuse to the next binding zone (Figure 3). However, even with this 4 nm displacement the probability that the tethered head is within ±1 nm of the next binding site is very low (*p* = 0.008). These stationary distributions suggest that for a 14 amino acid neck linker modeled with a drift corresponding the force extension curve of the WLC, the force required to stretch the chain in the range of 3–5 nm is sufficiently high that diffusion to these extended distances is very rarely achieved.

These diffusive steps were integrated into the kinetic model shown in Figure 1, and motor velocity and run length were obtained through simulations of the full hydrolysis cycle using the kinetic parameters given in Table 1. The binding step that is being modeled (State 4 to State 1 transition in Figure 1) involves diffusion of the head to the binding site followed by microtubule attachment. Thus, the attachment rate constant when the head is in the ±1 nm binding zone, *k_attach_* must be chosen empirically to achieve an effective attachment rate that is faster than the overall motor stepping rate of ∼100 s^−1^. Hence, *k_attach_* was set to 7.5×10^4^ s^−1^. Note that this is a first-order rate constant, with the probability of being within the binding zone accounting for the concentration term. While this rate constant appears fast, the relative concentration of one motor in a hemispheric volume of radius 1 nm around the binding site is 0.8 M, so the equivalent bimolecular on-rate is ∼10^5^ M^−1^s^−1^ (also see Discussion). As seen in Table 2, the predicted Kinesin-1 velocity (860±9 nm/s, mean ± SEM, N = 50 runs) and run length (1541±198 nm) agreed well with the experimentally observed values of 703±136 nm/s and 1747±199 nm [17], respectively.

**Table 1:** Rate constants used in chemomechanical model.

Rate Constant	Value
*k_detach_*	250 s^−1^
*k′_detach_*	0.25 s^−1^
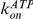	3 µM^−1^s^−1^
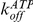	200 s^−1^
*k_hydrolysis_*	300 s^−1^
*k′_hydrolysis_*	8 s^−1^
*k_attach_*	WLC_Lp = 0.7nm_: 75,000 s^−1^
	WLC_Lp = 2nm_: 12,000 s^−1^
	Hookean: 10,000 s^−1^
	Reflecting: 3,500 s^−1^
*k′_attach_*	0.45 s^−1^
*k_unbind_*	3 s^−1^

Final parameters used in the stochastic simulations of Kinesin-1 and Kinesin-1_+DAL_ motors using the model shown in Figure 1. Model and rate constants were adapted from Muthukrishnan et al. [17]. The value of *k_attach_*, the attachment rate when the tethered head is within 1 nm of the binding site, depended on which neck linker model was used and was set to zero if the free head was more than 1 nm from the microtubule binding site. *k′* denotes reverse rate constants.

**Figure 4:**
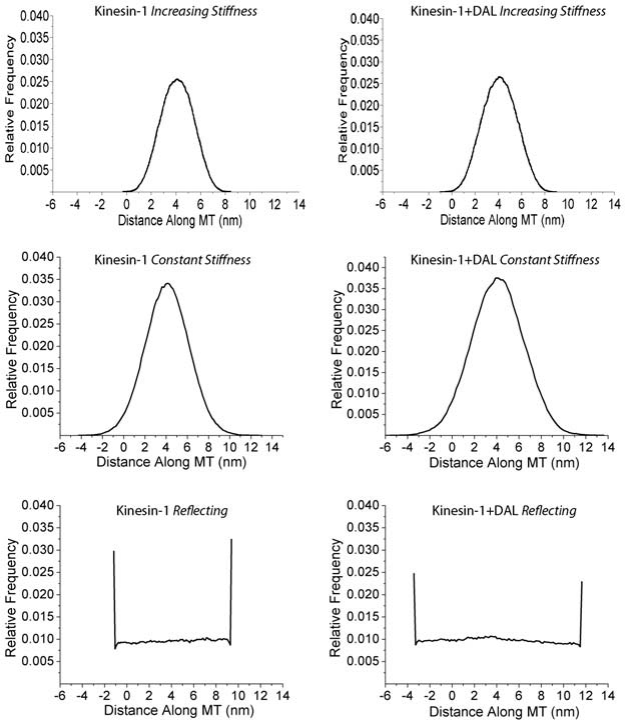
Stationary Distributions for All Models. Stationary distributions for the *Increasing Stiffness*, *Constant Stiffness*, and *Reflecting* models of the neck linker domain. The position of the free head of Kinesin-1 and Kinesin-1_+DAL_ was simulated by setting *k_attach_* to zero. A 4 nm bias resulting from ATP binding and neck linker docking is assumed in all cases.

To test the validity of the *Increasing Stiffness Model*, we simulated the behavior of a Kinesin-1 motor containing a three amino acid insert in the neck linker domain, Kinesin-1_+DAL_. These three residues correspond to the last three residues in the Kinesin-2 neck linker domain, which is 17 amino acids compared to 14 for Kinesin-1. In recent single molecule experiments, Kinesin-1_+DAL_ was shown to move at 552±103 nm/s, slightly slower than wild-type, and have a run length of 355±14 nm, which is more than four-fold shorter than wild-type [17]. Compared to wild-type Kinesin-1, the stationary distribution for Kinesin-1_+DAL_ is significantly broadened (Figure 4), meaning intuitively that under the increasing force model the motor spends a larger proportion of its time within 1 nm of the binding zone. As a result, when the diffusive step was integrated into the entire kinetic model, simulations predicted a moderate increase in the mean velocity to 944±10 nm/s and a significant increase in the run length to 3707±469 nm (Table 2). Inspection of the model makes this clear – State 4 is a vulnerable state and increasing the effective attachment rate (equal to k_attach_ multiplied by the fraction of time the head spends in the binding zone) decreases the probability of the motor detaching from that state. Similar behavior was observed when the persistence length in the *Increasing Stiffness Model* was increased from 0.7 nm to 2 nm (Table 1).

**Table 2:** Velocity and run length results.

	Kinesin-1		Kinesin-1_+DAL_	
	Run Length(nm)	Velocity(nm/s)	Run Length(nm)	Velocity(nm/s)
**Experimental ** **Results**	1747±199 (57)	703±136 (58)	355±14 (136)	552±103 (97)
***Increasing Stiffness Model*** ** (** ***L_p_*** ** = 0.7 nm)**	1541±198	860±9	3707±469	944±10
***Increasing Stiffness Model*** ** (** ***L_p_*** ** = 2.0 nm)**	1462±149	861±10	2477±374	934±11
***Constant Stiffness Model***	1915±247	860±6	2070±277	917±15
***Reflecting Model***	1777±238	858±8	1346±221	800±17

Experimental run length (mean ± SE of exponential fit (N)) and velocity (mean ± SD (N)) of Kinesin-1 and Kinesin-1_+DAL_ were taken from bead assays at saturating ATP (1 mM) from Muthukrishnan et al. [17]. Run lengths and velocities from simulations used the model structure shown in Figure 1 with parameters given in Table 1 and 1 mM ATP concentration. Run length (nm) and velocity (nm/s) reported as mean ± SEM for 50 runs each.

### Constant Stiffness Model

In an attempt to better account for the experimentally observed reduction in the velocity and run length of Kinesin-1_+DAL_, the *Increasing Stiffness Model* was simplified to a *Constant Stiffness Model* corresponding to a Hookean spring. As seen in Figure 2B, the Hookean spring has a much more liberal force-extension curve than the *Increasing Stiffness Model* and is predicted to allow the motor to diffuse to the binding site much more readily. A spring stiffness of 1 pN/nm was chosen, which is comparable to the observed entropic elasticity of disordered polypeptides during extension [27], [37]. Kinesin-1_+DAL_ neck linkers were modeled by adjusting the spring stiffness to a value of 0.8 pN/nm to reflect the increase in length from 14 to 17 amino acids. For the *Constant Stiffness Model*, which is represented by a linear stochastic differential equation, transition probabilities are Gaussian allowing for an exact simulation on the discrete time steps.

As seen in Figure 4, the stationary positional distribution of the free head for both Kinesin-1 and Kinesin-1_+DAL_ were significantly broader for the *Constant Stiffness Model* than for the *Increasing Stiffness Model*, meaning that the free head has a higher probability of existing within 1 nm of the binding site (*p* = 0.058). Setting *k_attach_* to 10^4^ s^−1^ resulted in a velocity of 860±6 nm/s and run length of 1915±247 nm for Kinesin-1, consistent with experimental data (Table 2). The velocity and run length values for Kinesin-1_+DAL_ were slightly elevated, which, like the *Increasing Stiffness Model*, is inconsistent with the experimental data.

### Reflecting Model

While the *Constant Stiffness Model* significantly reduced the constraints on the diffusion of the free head, it is physically unreasonable to predict that the neck linker domain will stretch beyond its maximum contour length. Hence, the third neck linker model examined included constant stiffness up to a reflecting barrier, which broadly corresponds to a Finitely Extensible Nonlinear Elastic (FENE) model having a small stiffness [35], [38]. Conceptually, the reflecting model is similar to rectified Brownian movement and is described by a reflected diffusion process with a strict upper and lower bound [39]. Quantitatively, the *Reflecting Model* combines a loose Hookean spring (*f_Spring_*) with barriers set by the contour length of the neck linker. The force-extension profile of the *Reflecting Model* is shown in Figure 2C, and the position of the motor head can be described as:
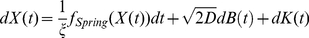
(3)We implement the model as a reflected diffusion (see [40] for an accessible introduction to reflected diffusion processes). Intuitively, if the diffusive forces on the motor head are sufficient to pass the limiting barriers during any time step, then the location of the motor head is constrained by the term *K*(t) to stay within the boundaries [35], [41]. At each time step, a numerical solution to Equation 3 is obtained by using Lepingle's adapted Euler method for reflected diffusions [41]. Lepingle's method uses a reflected Brownian motion approximation to the diffusion process near the barriers preventing an excess number of values at the boundary. The limiting barriers were positioned at a distance equal to the contour length of the tethering neck linker away from the anchor point of the spring (5.3 nm for Kinesin-1). Analysis of positional distributions using different spring constants revealed that a spring stiffness of ≤0.01 pN/nm allowed for the motor head to experience nearly unbiased diffusion (i.e. a flat distribution). The Kinesin-1 and Kinesin-1_+DAL_ stationary distributions using a *Reflecting Model* with a spring constant *k* = 0.01 pN/nm are shown in Figure 4.

Because diffusion of the free head is relatively unconstrained (within its maximal limits) in the *Reflecting Model*, the free head spends a significant fraction of its time (*p* = 0.18) within ±1 nm of the binding site, and a *k_attach_* of 3,500 s^−1^ is sufficient to achieve an effective attachment rate that is faster than the overall stepping rate. When this diffusional model was integrated into the entire kinetic cycle, the Kinesin-1 simulations (858±8 nm/s velocity and 1777±238 nm run length) again agreed with experimental data. More importantly Kinesin-1_+DAL_ had a slightly reduced motor velocity (800±17 nm/s) and a run length (1346±221 nm) that was shorter than wild-type (Table 2). This result qualitatively agrees with the experimental data – extending the neck linker domain reduces the motor run length. This reduction in the Kinesin-1_+DAL_ run length can be understood by examining Figure 4 – extending the Kinesin-1 neck linker effectively expands the region over which the free head diffuses, thus decreasing the proportion of time the motor spends within 1 nm of the binding zone. Using an identical *k_attach_* leads to a slower effective attachment rate and increases the probability of detachment during each diffusive step.

## Discussion

Mechanistic models describing the directed movement of molecular motors can involve concerted conformational changes, Brownian motion, or a combination of these mechanisms. For Kinesin-1, a body of experimental data supports the idea that ATP binding docks the neck linker of the bound head and displaces the free head toward the next binding site. However, to complete the step the free head must diffuse to its binding site, bind there, and release its bound ADP to achieve a high affinity microtubule-bound state (Figure 1). Because the free head is tethered during this diffusive step, the mechanical properties of the neck linker domain play an important role. If the neck linker is too short and/or too stiff, then the free head cannot reach the next binding site. However, if the neck linker domain is too long and/or too compliant, then the inter-head tension will be insufficient to coordinate the chemomechanical cycles of the two heads (front-head and rear-head gating) [4]. The need for investigating the role of the neck linker domain in tethered diffusion is of particular importance for understanding recent studies that have shown that artificially extending the Kinesin-1 neck linker profoundly affects motor behavior [8], [17], [42]. Because neck linker domains in diverse members of the kinesin superfamily diverge in sequence and length, understanding neck linker dynamics will also help to uncover how different kinesins are evolutionarily tuned to their specific cellular functions.

Here, we model the free kinesin head as a sphere and the microtubule as a one-dimensional lattice of binding sites, and we investigate the diffusion of the free head tethered by different qualitative representations of the flexible neck linker domain. Because the WLC is the most commonly used model to describe the force-extension characteristics of unstructured polypeptides, our analysis initially focused on the *Increasing Stiffness Model*. The striking result is that due to the stiffness of the neck linker, the diffusing free head spends only a small fraction of the time (*p* = 0.008) near its binding site, and thus extending the neck linker domain is expected to increase the processivity, contrasting with experimental results.

The first question to address is whether the fast attachment rate (*k_attach_* = 7.5×10^4^ s^−1^) needed to reproduce the experimental Kinesin-1 velocity and run length results using the *Increasing Stiffness Model* is realistic. While this first-order on-rate is consistent with a reasonable bimolecular on-rate of ∼10^5^ M^−1^ s^−1^, achieving tight binding to the microtubule also requires ADP release, which is thought to occur at a rate slower than 10^3^ s^−1^ [22], [42]. Without this tight binding resulting from ADP release, the head would rapidly unbind and diffuse back toward its resting position, significantly slowing down the process. Furthermore, extending the model to three dimensions would amplify this discrepancy – if the probability of being within ±1 nm of the binding site is 0.008 in one dimension, then it would drop to <10^−6^ in three dimensions. Because the effective attachment rate is equal to *k_attach_* multiplied by the probability the head is within 1 nm of its binding site, a 10^−6^ probability would require a *k_attach_* greater than 10^8^ s^−1^ to achieve a 100 s^−1^ overall motor stepping rate. Hence, in the *Increasing Stiffness Model* there is a significant discrepancy between the fast attachment rate needed for the model to work and the observed ADP release rate, which is the step that regulated tight binding of the head to the microtubule.

The second and more fundamental argument against the *Increasing Stiffness* model is that it predicts that mutations that extend the Kinesin-1 neck linker will enhance both motor velocity and processivity, which is the opposite of what is seen experimentally [8], [17], [42]. This point deserves closer inspection. State 4 is a vulnerable state in the kinesin hydrolysis cycle because following ATP binding and hydrolysis there is a competition between binding of the tethered head and unbinding of the attached head. Due to this kinetic bifurcation, any mechanism that slows down the attachment step without altering the unbinding step will increase the probability of detachment and therefore reduce the overall run length. Quantitatively, the probability of detaching per step is equal to 
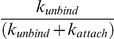
, so this dependence holds true even if this attachment step is nowhere near rate limiting. Importantly, using the chemomechanical cycle shown in Figure 1, any neck linker model that includes non-negligible restoring forces will predict an increase in motor processivity when the neck linker domain is extended. This includes the *Increasing Stiffness Model* using a 2 nm persistence length (Table 2) and the *Constant Stiffness Model*, and it would also be expected for polymer models such as a Freely Jointed Chain. The reason is that in all of these models, extending the neck linker increases the probability that the tethered head will be near its binding site, which increases the effective attachment rate. While it can't be ruled out that extending the neck linker alters other rate constants in the chemomechanical cycle, because no other steps are as intimately linked to motor processivity, it is unlikely that these compensating changes can resolve the discrepancy between experimental results and the *Increasing Stiffness Model* simulations.

In contrast to the *Increasing Stiffness Model*, when the neck linker was modeled as a reflecting process, the free head spent a significant fraction of its time within ±1 nm of the next binding site. Hence, achieving a reasonable effective attachment rate only required a *k_attach_* of 3,500 s^−1^, which is closer to experimentally measured ADP release rates [22], [42]. Furthermore, extending the neck linker predicted a decrease in both motor velocity and run length, consistent with experimental results. The drawback to the *Reflecting Model* is that it ignores any entropic spring characteristics of the flexible neck linker and instead assumes an extremely compliant neck linker domain up to the maximum limits of extension. Quantitatively, a Worm-Like Chain with *L_p_*>*L_c_* achieves this same force-extension profile, but because the WLC approximation was developed for polymers with *L_c_*>*L_p_* and ignores any compressive forces, this comparison should be treated cautiously.

**Figure 5:**
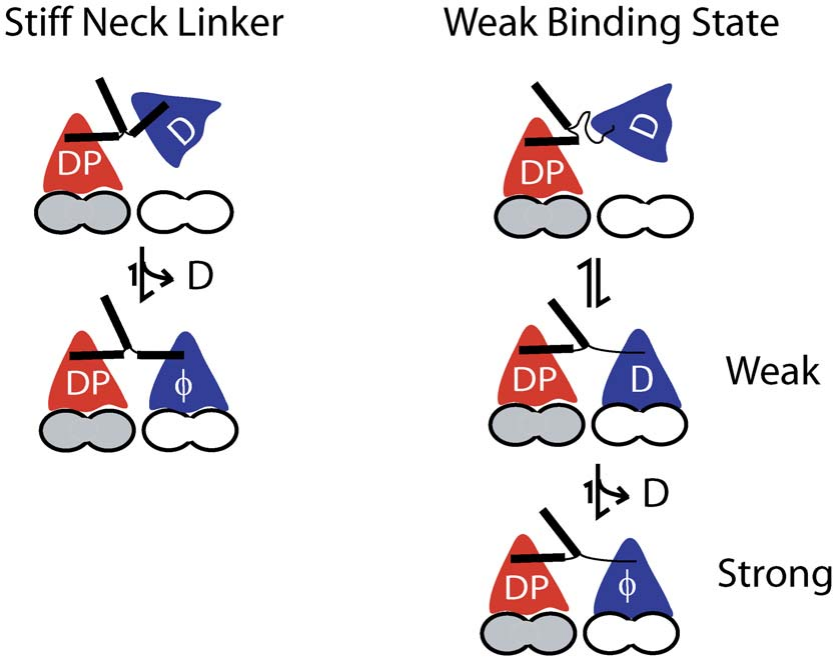
Possible Resolutions to the *Increasing Stiffness Model*. If the neck linker is considerably stiffer than estimated from WLC models (perhaps stabilized through interactions with the core motor domain), then it would act more like a pivoting rod. Thus, the tethered head would diffuse in the vicinity of its binding site. Extending the neck linker would be expected to position the tethered head beyond its binding site, slowing the rate of attachment. Alternatively, the tethered head may be stabilized near its microtubule binding site by weak electrostatic interactions with the microtubule that counteract the restoring force of the neck linker tether. ADP release would then trigger strong binding to the microtubule.

How is it possible to reconcile the *Increasing Stiffness Model* simulations, which suggest that the neck linker strongly limits diffusion of the free head, with the more experimentally consistent *Reflecting Model* results that rely on a physically improbable model of the neck linker domain? There are two possible resolutions to this conflict. The first possibility is that the undocked neck linker is actually much stiffer than predictions from the WLC (Figure 5). A 14 or 17 amino acid polypeptide is considerably shorter than polymers such as titin that have been experimentally measured and successfully fit with the WLC model [24], [25], [30], [31]. While the Molecular Dynamics simulations presented in Figure 2 suggest that the Kinesin-1 neck linker properties are reasonably well fit by the WLC, these simulations did not include other regions of the motor domain that may help to stabilize the neck linker in a more extended conformation. It should be noted that in a crystal structure of the mitotic motor Eg5 (Kinesin-5) in ADP, the neck linker interacts stably with the head in a perpendicular position [43]. This suggests that the neck linker in the leading head would be relatively straight and stabilized and would not act as a flexible tether at all. An analogous neck linker position for Kinesin-1 was observed by Rice et al (Figure 4d in [5]), although key residues that stabilize this conformation in Kinesin-5 are absent in Kinesin-1. Nonetheless, if the neck linker domain were considerably stiffer as a result of this docking mechanism or some other structural feature, then it would act more as a pivoting rod and the tethered head would spend considerably more time near the next binding site. Neck linker extensions would then be expected to have slower attachment rates because the head is “pushed” beyond its optimal position. In principle, this hypothesis could be tested by attaching fluorescent probes to either end of the neck linker domain and monitoring its end-to-end length by fluorescence resonance energy transfer.

A second way to resolve the models is to posit a weak binding state preceding ADP release of the tethered head (Figure 5). Despite the head residing near the binding site less than 1% of the time in the *Increasing Stiffness Model* simulations, the recurrence time (mean time to return to the binding zone after leaving) is still under one microsecond (350 nsec for the *Increasing Stiffness Model*). Hence, the kinetics of reaching the binding site are not at all limiting, and instead the problem is that the head rapidly diffuses away from this extended position before having a chance to bind. If there were a stabilizing interaction between the head and the microtubule (a weak binding state), such that the head was held at this extended position, this would increase the fraction of time the head remained in the binding zone and hence increase the probability that ADP was released to trigger tight binding. Positive charge in the kinesin motor domain, neck linker domain, and neck coil domain have all been shown to enhance processivity [18], [44], [45]. Such a weak binding state for kinesin has been proposed by Cross (M·K^TRAPPED^·ADP) [16], and similar weak binding states have been characterized in myosin [46]. For this weakly-bound state to facilitate ADP release and thus resolve this kinetic disparity, it would need to significantly shift the equilibrium to the bound state against the restoring force of the extended neck linker; however this interaction couldn't be too tight or it would slow the subsequent detachment of the head during the next step (i.e. *k_detach_* in Figure 1). Because this weak-binding conformation would be expected to be stabilized by electrostatic interactions between the kinesin head and the microtubule, this hypothesis could in principle be tested by introducing mutations in the microtubule binding site and/or increasing the ionic strength and measuring whether the processivity is diminished.

By integrating tethered diffusion into a chemical kinetic model of the kinesin hydrolysis cycle, we find that restoring forces imposed by the flexible neck linker domain profoundly constrain the ability of the free head to diffuse to its binding site. When the neck linker domain is modeled as a spring with a length-dependent stiffness (a WLC), the required attachment rates for Kinesin-1 are very high and the predicted behavior of motors with extended neck linkers contrasts with experimental results. The present modeling work suggests that either a) the neck linker domain is very compliant up to an inextensible limit (*Reflecting Model*), b) the neck linker resides in a more extended conformation than is generally thought, perhaps stabilized by the core motor domain, or c) stabilizing interactions between the tethered free head and the microtubule (a weak binding state) hold the tethered head in place to allow ADP release and strong binding that completes the motor step. These hypotheses can be tested by structural and kinetic analysis of wild-type and mutant kinesins, as well as by comparing the behavior of diverse motors across the kinesin superfamily.

## Methods

We numerically simulated the processive stepping of single, homodimeric kinesin motors, incorporating the diffusion of the free motor head to its next binding site on the microtubule. Figure 1 outlines the chemomechanical cycle of the motor. For each state transition, the directionality and dwell times are stochastically determined using a continuous time, discrete space Markov chain that may depend on the position of the unbound motor head. In states that do not include free head diffusion (State 1), motor transitions and dwell times are determined using the Gillespie Stochastic Simulation Algorithm [47]. Dwell times are exponentially distributed with a mean time equal to 1/Σ*k_1j_*, independent of a forward or backward transition.

States modeling one bound head and one freely diffusing head require the additional computation of the position of the free head. In these states, the free motor head is diffusing about the center point of a potential determined by the neck linker model. The position of the motor head, *X(t)* is modeled using the over-damped Langevin equation, representing the neck liner tether (*f_tether_*) and Brownian forces on the free head [34], [35]. The motor head was represented by a sphere with a radius, *a* = 3 nm where the drag coefficient, *ξ*, was calculated as the Stokes friction coefficient of a sphere with viscosity of water *η* = 10^−9^ g/nm s [48], [49]:
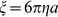
(4)Brownian forces on the motor head were interpreted as an Ito differential [36] yielding:
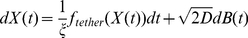
(5)where *D = k_B_T/ξ* and *B(t)* is a standard Wiener process An intuitive way to think about Equation 5 is as:
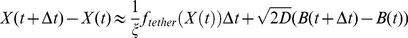
(6)thus the last term is a Gaussian random variable with mean zero and variance *2DΔt*. A discrete time Euler approximation was used to model the state transition time, where the current position of the diffusing motor head was obtained by a numerical integration of the Langevin equation (specific to each neck linker model and described in the results section). When the motor transitions between chemical states 2, 3 and 4, the free head is diffusing according to the equation for that particular state; however, the initial value of the diffusion for one state is determined by the location of the free head at the previous state. For example, the location of the free head does not change when moving from chemical state 2 to state 3, but the equations governing the continuous dynamics do change.

For a given time step, whether a chemical transition or binding event occurs is determined by a Bernoulli random variable and with a probability determined by the binding rate times the time step of the simulation. In diffusive states 2 and 3, state transitions do not depend on the position of the motor head, however in state 4 the probability of binding is dependent on the current position of the free motor head. For transitions between rear and forward diffusing motor domains, the center point of the neck linker potential is shifted in the respective direction. ATP-dependent neck linker docking was incorporated into the model by introducing a 4.1 nm positional bias toward the microtubule (+) end following ATP binding (States 3 and 4). In these ATP bound states, the tether consisted of only one neck linker domain (that of the free head).

In the diffusive binding state (State 4), defined microtubule sites at a distance 8.2*n (where *n* = −1, 1) nanometers relative to the bound motor domain allow for multiple binding options during a diffusive search. A region of ±1 nanometer was designated about each binding site in which the diffusing motor head could attach to the microtubule with a fixed first-order rate constant *k_attach_*, and binding was prohibited if the head was outside this 7.2–9.2 nm binding zone (Figure 2A). Upon binding, the free head was placed at the center of the binding site 8.2*n nanometers away from the bound head.

### Sketch of Algorithm

To make this description more concrete, we present a sketch of the algorithm used for simulation. The description below details the conditions required to transition through each of the four chemical states of a full cycle that comprises a single mechanical step. The full algorithm requires keeping track of each individual head and the distance each moves while free.

State 1: Both Heads Bound.

1. Simulate an exponential hold time with rate *k_detach_+k′_detach_*.
2. Simulate a uniform random variable, *rand*. Move to State 2 with probability *k_detach_/(k_detach_+k′_detach_)* and State 4 with probability *k′_detach_/(k_detach_+k′_detach_)*.

State 2: Initial condition for head is set to location of binding site, −8.2 nm. Potential is centered between binding sites at −4.1 nm. Set time in State 2: *t* = 0, *n* = 0. Set attachment rate *k(x) = k_attach_* if head is within 1 nm of either forward or rearward binding site, otherwise *k(x)* = 0.

1. Solve Langevin equation for fixed time step Δ; find *X_n+1_* from *X_n_*. Set *t* = *t*+Δ, *n* = *n*+1.
2. Check for binding or reaction; generate a uniform random number, *rand*.
  - If 0≤*rand*≤*k(X_n_)*Δ, then move back to chemical State 1.
  - If *k(X_n_)*Δ<*rand*≤(*k(X_n_)*+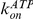)Δ, then move to chemical State 3.
  - If *rand*>(*k(X_n_)*+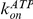)Δ, remain in State 2 and return to step 1.

State 3: Initial condition for head is determined by the terminal location of the free head from the previous chemical state (2 or 3). Center of the potential is moved to the location of the bound head (0 nm). Set time in State 3: *t* = 0.

1. Solve Langevin equation for fixed time step Δ; find *X_n+1_* from *X_n_*. Set *t* = *t*+Δ, *n* = *n*+1.
2. Check for binding or reaction; generate a uniform random number, *rand*.
  - If 0≤*rand*≤
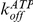
Δ, then move back to chemical State 2.
  - If 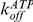
Δ<*rand*≤(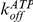
+*k_hydrolysis_*)Δ, then move to chemical State 4.
  - If *rand*>(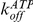
+*k_hydrolysis_*)Δ remain in State 3 and return to step 1.

State 4: Initial condition for head is determined by the terminal location of the free head from the previous chemical state (3 or 1). Center of the potential is moved to a position *x* = 4.1 nm forward of the bound head, corresponding to ATP-induced docking of the neck linker domain. Set time in State 4: *t* = 0. Define attachment rate *k(x) = k_attach_* if head is within 1 nm of next binding site, otherwise *k(x)* = 0.

1. Solve Langevin equation for fixed time step Δ; find *X_n+1_* from *X_n_*. Set *t* = *t*+Δ, *n* = *n*+1.
2. Check for binding or reaction; generate a uniform random number, *rand*.
  - If 0≤*rand*≤*k′_hydrolysis_*Δ, then move back to chemical State 3.
  - If *k′_hydrolysis_*Δ<*rand*≤(*k′_hydrolysis_+k_unbind_*)Δ, then move to chemical State 5 (released from microtubule).
  - If (*k′_hydrolysis_+k_unbind_*)Δ<*rand*≤(*k′_hydrolysis_+k_unbind_+k(X_n_)*)Δ, then move to chemical State 1.
  - If *rand*>(*k′_hydrolysis_+k_unbind_+k(X_n_)*)Δ, remain in State 3 and return to step 1.
